# Managing diabetes in people with dementia: protocol for a realist review

**DOI:** 10.1186/s13643-015-0182-4

**Published:** 2016-01-07

**Authors:** Frances Bunn, Claire Goodman, Jo Rycroft Malone, Peter Reece Jones, Chris Burton, Greta Rait, Daksha Trivedi, Antony Bayer, Alan Sinclair

**Affiliations:** Centre for Research in Primary and Community Care, University of Hertfordshire, College Lane, Hatfield, Hertfordshire UK; School of Healthcare Sciences, Bangor University, Bangor, UK; Centre for Health-Related Research, Bangor University, Bangor, UK; Research Department of Primary Care and Population Health, UCL Medical School (Royal Free Campus), Rowland Hill Street, London, UK; Cochrane Institute of Primary Care and Public Health, Cardiff University, Cardiff, UK; Foundation for Diabetes Research in Older People, London, UK

**Keywords:** Dementia, Diabetes, Realist review

## Abstract

**Background:**

Worldwide, the prevalences of diabetes and dementia are both increasing, particularly in older people. Rates of diabetes in people with dementia are between 13 and 20 %. Diabetes management and diabetic self-care may be adversely affected by the presence of dementia. There is a need to know what interventions work best in the management of diabetes in people living with dementia (PLWD) in different settings and at different stages of the dementia trajectory. The overall aim is to develop an explanatory account or programme theory about ‘what works’ in the management of diabetes in people in what context and to identify promising interventions that merit further evaluation.

**Methods/design:**

This study uses a realist approach including studies on the management of diabetes in older people, medication management, diabetes-related self-care, workforce issues and assessment and treatment. We will use an iterative, stakeholder driven, four-stage approach. Phase 1: development of initial programme theory/ies through a first scoping of the literature and consultation with key stakeholder groups (user/patient representatives, dementia-care providers, clinicians, diabetes and dementia researchers and diabetes specialists). Phase 2: systematic searches of the evidence to test and develop the theories identified in phase 1. Phase 3: validation of programme theory/ies with a purposive sample of participants from phase 1. Phase 4: actionable recommendations for the management of diabetes in PLWD.

**Discussion:**

A realist synthesis of the evidence will provide a theoretical framework (i.e. an explanation of how interventions work, for whom, in what context and why) for practice and future research work that articulates the barriers and facilitators to effective management of diabetes in people with dementia. By providing possible explanations for the way in which interventions are thought to work and how change is achieved, it will demonstrate how to tailor an intervention to the setting and patient group. The propositions arising from the review will also inform the design of future intervention studies.

**Systematic review registration:**

PROSPERO registration number CRD42015020625.

**Electronic supplementary material:**

The online version of this article (doi:10.1186/s13643-015-0182-4) contains supplementary material, which is available to authorized users.

## Background and rationale

Dementia and diabetes mellitus are common long-term conditions and may co-exist in a large number of older people [1–3]. Worldwide, there are an estimated 35.6 million people with dementia. By 2050, this number will rise to over 115 million [4]. Although there are differences in the physical and cognitive effects of the different types of dementias, all are progressive, involve increasing physical and mental deterioration and lead to a person with dementia to become increasingly dependent. Diabetes mellitus is seen in 10–25 % of older people [5, 6], and in nursing homes, up to 27 % of residents may have diabetes [7–9]. As with dementia, the prevalence of type 2 diabetes is increasing globally [10, 11] and there is evidence to suggest there is a link between cognitive dysfunction and type 2 diabetes [1, 12, 13]. A recent scoping review found data to suggest that rates of diabetes in people with dementia are between 13 and 20 % [14].

Having dementia or cognitive impairment impacts on a person’s ability to understand their condition and undertake self-care management tasks such as managing medication and monitoring blood glucose [3, 15–17] and is associated with an increased use of both health and social services [17]. Moreover, people with dementia are at greater risk of hypoglycaemia than older people without dementia [18–21] and may be at risk of drug interactions and adverse reactions due to polypharmacy [22]. There is also evidence that people with dementia may have poorer access to diabetes services and monitoring than people without dementia [23–26].

There is currently no systematic approach to the management of diabetes and dementia [27], and most care pathways for diabetes do not take into account the needs of people with dementia [28]. Clinical guidance on the management of diabetes in older adults [22, 29–31] suggests that glycaemic targets should be individualised for older people and take into account factors such as age, dementia, frailty, comorbidities and polypharmacy [32, 33]. However, there is limited evidence on the outcomes of such approaches for people with dementia [34].

The main approach to the management of long-term conditions such as diabetes revolves around self-management strategies focusing on the attitudes and self-efficacy of the patient, for example, using motivational interventions [35] or education programmes [36, 37]. Whilst this may be appropriate in the earlier stages of dementia [38], capacity for self-management will diminish as the dementia progresses and interventions may instead have to target family carers. The situation is further complicated in that management may differ for those whose diabetes pre-dates a diagnosis of dementia compared to patients who develop diabetes post-dementia. There are additional difficulties related to insulin management. Interviews conducted as part of a recent National Institute for Health Research (NIHR) study suggest that as people living with dementia become unable to manage their own medication, they find injections distressing and painful [39]. They may also forget to eat or take medication leading to episodes of hypoglycaemia and in some instances hospitalisation [39].

Physical frailty [33] or end-stage dementia compound the complexity of diabetes management, with decisions needing to be made about whether to maintain treatment or consider admission into nursing home care [40]. The underlying assumption of this proposal is that the effectiveness of programmes to manage diabetes in people with dementia is contingent not only on specific diabetes-focused interventions but also on contextually situated decision-making. Interventions designed to improve the management of diabetes in people with dementia are likely to be multi-component, specific to different stages of the dementia trajectory and dependent on the behaviours and choices of those delivering and receiving the care.

To develop a theoretical understanding of the realities of working in and across complex, overlapping systems of care and why and how different interventions may work, there is a need to synthesise the different strands of research evidence. Realist synthesis is a systematic, theory-driven approach that aims to make explicit the mechanism(s) of how and why complex interventions are effective (or not) in particular settings [41–45]. Realist synthesis takes account of a broad evidence base as well as the experiential and clinical knowledge that relates to the physiology and management of diabetes in older people and specifically older people with dementia. Evidence of interest would include evaluations of interventions relating to glycaemic control in older people, medication management, diabetes-related self-care and those that address system-wide issues about access to assessment and treatment. It would also include those that, by association, have the potential to improve diabetes care for people with dementia (for example, studies on relationship-centred care, interventions to individualise or target care for people with dementia and the development and implementation of interventions). Realist synthesis methodology will enable us to deconstruct the component theories underpinning different interventions aimed at people with dementia and/or diabetes and to consider relevant contextual data to test our understanding of the applicability of different approaches for this population and in different settings.

## Methods

We will use an iterative four-stage approach that optimises the knowledge and networks of the research team. The review is based on the stages set out by Pawson et al. [46] and follows the RAMESES publication standards [45]. The objectives are to

1. Identify how interventions, or elements of interventions, to manage diabetes in people with dementia are thought to work, on what range of outcomes (i.e. organisational, resource use and patient care and safety) and for whom they work (or why they do not work) and in what context
2. Identify the barriers and facilitators to the acceptability, uptake and implementation of interventions designed to manage diabetes in people with dementia
3. Establish what evidence there is on the feasibility and potential value of interventions to manage diabetes in people with dementia
4. Establish what is known about the design of diabetes management technologies and identify the potential benefits of involving end users (people with dementia and their carers) in their development.

### Ethics

Ethical approval for the stakeholder interviews has been obtained from the University of Hertfordshire Health and Human Sciences Ethics Committee with delegated authority (ECDA), reference number cHSK/SF/UH/00106.

A PRISMA-P file is attached (Additional file 1).

### Realist approach

Within this review, the purpose is to develop an explanatory account or programme theory about ‘what works’ in the management of diabetes in people with dementia, and in what context. Explanatory accounts comprise configurations of context (the contingencies on which programme changes are dependent), mechanism (the changes that are brought about through a programme) and outcomes (programme impacts). These configurations are developed iteratively through data collection, theorising and stakeholder engagement.

We are working on the assumption that a review on the management of diabetes in people with dementia has to consider complementary evidence. For example, evidence on the effectiveness of interventions to improve diabetes management in older people without dementia, interventions to improve the health and well-being of people with dementia and studies that rely on healthcare professionals in different settings working together, and with family carers, to improve diabetes management for older people. It is likely that the review will be informed by theoretical work on the following:

- Management of diabetes in older adults and those with complex health needs (including issues such as individualising glycaemic targets for people with dementia, glycaemic targets that take into account the risk and impacts of hypoglycaemia in this group) [29, 30, 46, 47]
- Patient-centred approaches to glycaemic management in people with diabetes that balance treatment targets against quality of life and patient and carer preferences [48, 49] and that involve patients and carers in the development of interventions
- Theories about the way services are designed, delivered or implemented for people with dementia, e.g. work that clinical staff do in tailoring or co-constructing interventions to individuals, including case management approaches, shared care models and assistive technology [50]
- Theories around diagnostic or clinical overshadowing—for example, if the dementia is associated with behaviours that are challenging (e.g. aggression, agitation, psychosis) then dementia may become clinically dominant and detract from the management of conditions such as diabetes [51, 52]
- Interventions that provide education or support for the family carers of people with dementia to help them cope with the behavioural, psychological and emotional consequences of dementia [53–58]
- Theories on the provision of person-centred/relationship-centred care for people with dementia [59–62].

### Phase 1: defining the scope of the review: concept mining and theory development

The project team will draw on their collective experience in diabetes, dementia, older people’s health and realist methods to develop initial programme theories about diabetes management interventions. Programme theories are possible explanations for the way in which particular interventions are thought to work, and they describe the way in which change occurs because of an intervention [63].

We will undertake a preliminary scoping of a selection of key literature (e.g. relevant evaluations of interventions for people with dementia or interventions for frail older people with diabetes). References collated by the project team for recent work on diabetes and dementia [14, 27, 31] will be supplemented by key word searches, discussion with the wider project team and interviews or focus groups with stakeholders. The following key stakeholder groups have been identified:

1. Clinicians with a special interest in the management of diabetes in older people,
2. Providers of care in primary and secondary care (e.g. diabetes specialist nurses, GPs and other clinicians),
3. User representatives including recipients of care and their family carers and relevant diabetes or dementia charities
4. Academics and those involved in developing education and guidance for older people with diabetes
5. Dementia specialists from primary, secondary and tertiary care and the voluntary sector (e.g. old age psychiatrists, dementia specialist nurses and GPs with an interest in dementia).

Stakeholder interviews will be conducted using a topic guide and, with permission, digitally recorded.

This will be followed by a 1-day workshop where the project team will begin to identify common concepts and map and prioritise the theory identified from the searches and consultation. The process will also draw on the existing research and clinical experience of the research team and project advisory group. To ensure transparency of approach, and an audit trail, we will record group discussions and maintain structured field notes on suggestions and decision-making processes about which sources of evidence were linked to which strands of theoretical development [42]. An output from phase 1 will be a theoretical/conceptual framework, and associated candidate programme theories, that will inform the review process.

### Phase 2: retrieval, review and synthesis

First, we will target evidence relevant to the management of diabetes in people living with dementia. This will include interventions that address the knowledge and skills required to promote effective diabetes care and specific interventions to manage diabetes in people with dementia or cognitive impairment (including those that focus on family carers). However, previous and current work by the project team suggests that there are few studies that look specifically at the management of diabetes in people with dementia or that evaluate interventions designed specifically for this population. Realist synthesis enables the testing of the relevance and rigour of emerging findings from one body of literature to another, and in line with the iterative nature of realist synthesis methodology [64], the inclusion criteria will be refined in light of emerging data and the theoretical development in phase 1.

The review is likely to include evidence sources that cover the following:

- People with mild, moderate or advanced dementia (of any type, e.g. Alzheimer’s disease, vascular dementia, Lewy body dementia, Parkinson’s disease dementia, fronto-temporal dementia and alcohol-related dementia) and type 1 or type 2 diabetes, resident in the community or a care home or other long-term setting, or who are being treated in hospital.
- Studies of any intervention designed to promote the management of diabetes in people with dementia and the prevention of potential adverse effects associated with poorly managed diabetes such as falls, blindness, vascular complications or renal failure.
- Studies that provide evidence on barriers and facilitators to the implementation and uptake of interventions designed to improve the physical health of people with dementia (e.g. dementia-friendly initiatives, the impact of the cognitive versus behavioural and psychological symptoms of dementia and the progression of dementia on family carers and service providers).
- Studies that offer opportunities for transferable learning such as those that evaluate interventions for people with dementia and other clinical conditions, or those that look at the way services are delivered and implemented for people with dementia (for example, interventions to: improve access or continuity, tailor care to the needs of individuals with dementia or support family carers).

### Outcomes

Outcomes will be established by the project team in an iterative process but are likely to include the following:

- Health and well-being of older people with diabetes and dementia and their family carers, e.g.
  - Glycaemic management and the prevention of hypoglycaemia and hyperglycaemia
  - Management of cardiovascular risk factors such as hypertension and hyperlipidemia
  - Identification, management and prevention of long-term complications such as depression, visual problems and neuropathic complications [28]
  - Medication adherence
  - Knowledge and quality of life for older people with dementia and their family carers.
- Outcomes related to service use, e.g. unplanned hospital admissions.
- Process-related outcomes, e.g. quality of care and ‘what works’ in terms of designing and tailoring diabetes management technologies.

### Types of studies

A diversity of evidence provides an opportunity for richer data mining and theory development. Therefore, we will include studies of any design including randomised controlled trials, controlled studies, uncontrolled studies, interrupted time series studies (ITS), cost effectiveness studies, process evaluations, surveys and qualitative studies of participants’ views and experiences of interventions. We will also include unpublished and grey literature, policy documents and information about locally implemented programmes in the UK.

### Searching for relevant studies

We will develop search strategies in the following areas: (1) dementia and diabetes (focusing on issues relating to management), (2) diabetes management in older adults and those with multimorbidity, (3) interventions to support people with dementia and comorbid health conditions (all conditions not just diabetes) and (4) interventions that involve the family carers of people with dementia. The search will be iterative, and search areas will be revised as the review progresses.

In conjunction with an information scientist, the project team will develop a list of relevant search terms to use in the following electronic databases: MEDLINE (PubMed), CINAHL (Cumulative Index to Nursing & Allied Health Literature), Scopus, Cochrane Library (including the Cochrane Database of Systematic Reviews, Database of Abstracts of Reviews of Effects (DARE)), the HTA Database, NHS Economic Evaluation Database (NHS EED), AgeInfo (Centre for policy on Ageing—UK), OpenGrey, Social Care Online, the National Research Register Archive, the National Institute of Health Research portfolio database, NHS Evidence and Google Scholar.

Dementia reviews undertaken by members of the project team [65, 66] have highlighted the importance of lateral searching for identifying studies in this area. Therefore, in addition to the above electronic database searches, we will undertake the following:

- Check reference lists from primary studies and systematic reviews [67]
- Perform citation searches using the ‘Cited by’ option on Google Scholar and Scopus, and the ‘Related articles’ option on PubMed [68]
- Contact experts and those with an interest in dementia to uncover grey literature (e.g. DeNDRoN, National Library for Health Later Life Specialist Library, Alzheimer’s Society and For Dementia)
- Contact diabetes-specific charities and user groups (Diabetes UK, TREND)

Search results will be downloaded into bibliographic software and, where possible, duplicates deleted. Two reviewers will independently screen titles and abstracts for relevance.

## Review

### Screening and data extraction

Two reviewers will screen full manuscripts for inclusion based on the relevance and rigour of the evidence, with disagreements resolved by discussion with a third author. Relevance is defined as the extent to which it can contribute to theory building and/or testing and rigour the extent to which the methods used to generate that particular piece of data are credible and trustworthy [41, 45]. For studies that meet the test of relevance, data will be extracted onto bespoke data extraction forms which will enable us to populate the evidence on context mechanism outcome configurations [44]. The data extraction form will be informed by programme theories that emerge from phase 1 and will be pre-tested by the review team [44]. Data will be extracted by one reviewer and checked by a second.

### Synthesis

The analytical task is in synthesising, across the extracted information, the relationships between mechanisms (e.g. changes in reasoning or resources), contexts (e.g. conditions, types of setting, organisational configurations) and outcomes (i.e. intended and unintended consequences and impact). Rycroft-Malone et al. [44] have developed an approach to synthesis, incorporating the work of Pawson [41] and principles of realist enquiry that includes the following:

1. Organisation of extracted information into evidence tables representing the different bodies of literature (e.g. diabetes management, dementia, health care, organisation of services, technology)
2. Theming across the evidence tables in relation to emerging patterns (demi-regularities in realist literature) amongst context, mechanism and outcomes (C-M-Os), seeking confirming and disconfirming evidence within studies and across the body of evidence as a whole.
3. Linking these demi-regularities (patterns) to refine hypotheses.

Data synthesis will involve individual reflection and team discussion and will (1) question the integrity of each theory, (2) adjudicate between competing theories, (3) consider the same theory in different settings and (4) compare the stated theory with actual practice. Data from the studies will then be used to confirm, refute or refine the candidate theories. If our theories fail to explain the data, we will seek alternative theories.

Once the preliminary mapping of the evidence is complete, we will hold a second 1-day workshop with the research team. This workshop will be structured to include in-depth discussion of the findings and to develop and confirm the resultant hypotheses. These will act as synthesised statements of findings around which we can develop a narrative that summarises the nature of the context, mechanism and outcome links, and the characteristics of the evidence underpinning them.

The transparency of a realist review synthesis is reliant on careful documentation of the reasoning processes, how they are grounded in the evidence and justification of inferential shifts through engagement with different evidence sources [42]. This aspect of the review process is resource intensive and reliant on discussion and deliberation, across and with particular members of the research team. Outputs from phase 2 will include a comprehensive evidence base related to programmes designed to manage diabetes in people with dementia and a set of hypotheses supported by relevant evidence to be refined in phase 3.

### Phase 3: test and refine programme theory/ies (validation)

To develop a final review narrative that addresses what is necessary for the effective implementation of programmes to manage diabetes in people with dementia, we will review the hypotheses and supporting evidence through telephone interviews with up to 15 stakeholders. Participants will be purposively sampled to ensure that all the key stakeholder groups in phase 1 are represented. A topic guide will be developed that aims to elicit stakeholder’s views on the resonance of the findings, both from a practice and a service user perspective.

### Phase 4: actionable recommendations

We will work with the project advisory group and commissioners and providers, e.g. CCG representatives and diabetes and dementia experts, to develop a set of actionable recommendations designed to inform practice and the development of future research studies. This will include the development of an evidence informed framework of what works for whom and in what context in relation to programmes to manage diabetes in people with dementia, and recommendations for promising interventions for future evaluation.

## Discussion

Dementia and diabetes mellitus are common long-term conditions and may co-exist in a large number of older people [1–3]. People with dementia may be less able to understand and manage their diabetes [3, 16, 17] and may be at risk of complications such as hypoglycaemic episodes, cardiovascular conditions and amputations which place a huge burden on health and social care economies [69]. Moreover the impact of diabetes and dementia on patients and their families is considerable. A realist synthesis of the evidence will provide a theoretical framework (i.e. an explanation of how interventions work, for whom, in what context and why) for practice and future research work that articulates the barriers and facilitators to effective management of diabetes in people with dementia. By providing possible explanations for the way in which interventions are thought to work and how change is achieved, it will demonstrate how to tailor an intervention to the setting and patient group. The propositions arising from the review will also inform the design of future intervention studies.

## Additional file

Additional file 1:
**PRISMA checklist DIaMonD.**
